# Treatise on the Dental Art, Founded on Actual Experience

**Published:** 1843-03-04

**Authors:** 


*Treatise on the Dental Art, founded on Actual Experience. Illustrated by two hundred and forty-one figures in Lithography, and forty-four Wood Cuts.* By F. Maury, Dentist of the Royal Polytechnic School. *Translated from the French, with Notes and Additions.* By J. B. Savier, Doctor in Dental Surgery. Philadelphia: 1843, 8vo. pp. 285.

M. Maury stands well as a Dental Surgeon in Paris, and the present treatise sustains an excellent reputation. It is divided into three parts. The first part treats of dental anatomy, physiology, and pathology. It is to be regretted that in the sections which treat of the structure and developement of the teeth, no notice should be taken by the translator of the now received views of Retzius, Owen, Nasmyth and Goodsir. Part second embraces the important subjects of dental hygiene and therapeutics, and contains much of interest and usefulness. The third and last part is devoted to Mechanical Dentistry, or Odontotechny, and the mechanical means of remedying lesions of the palatine arch. This portion was defective in the original work, and the deficiency has been supplied from the Treatise of Dr. Solyman Brown. An alphabetical list of writers on Dental Science is appended.

"The translator has endeavoured to give a faithful interpretation of the views of his author, and would have preferred essaying no further than this, did not the many improvements made in this department of the curative art, within a few years past, render it necessary that he should omit or condense certain passages, that would in the present condition of the science be of no profit, and to append notes to others, to explain or refute whatever appeared ambiguous or erroneous."—*Preface p.* v.

To the practitioner of dentistry, and to the country physician, the present work will be a valuable acquisition. By an acquaintance with dental pathology and therapeutics, the latter might save much suffering, and in many instances subsequent permanent inconvenience.

